# Navigating and manipulating childbirth services in Afar, Ethiopia: A qualitative study of cultural safety in the birthing room

**DOI:** 10.1016/j.socscimed.2023.116073

**Published:** 2023-08

**Authors:** Ashley Hagaman, Humberto Gonzalez Rodriguez, Emilie Egger, Befikadu Bitewulign, Haley Case, Abiyou Kiflie Alemayehu, Elizabeth C. Rhodes, Abiy Seifu Estifanos, Kavita Singh, Dorka Woldesenbet Keraga, Mahrukh Zahid, Hema Magge, Clare Barrington

**Affiliations:** a Department of Social and Behavioral Sciences, Yale School of Public Health, Yale University, 60 College St, New Haven, CT, 06510, USA; b Center for Methods in Implementation and Prevention Sciences, Yale University, New Haven, CT, USA; c Department of Health Behavior, Gillings School of Global Public Health, University of North Carolina at Chapel Hill, 135 Dauer Dr., Chapel Hill, NC, 27599, USA; d Institute for Healthcare Improvement, Addis Ababa, Ethiopia; e Centers for Disease Control and Prevention 1600 Clifton Road, Atlanta, GA, 30329, USA; f Hubert Department of Global Health, Rollins School of Public Health, Emory University, 1518 Clifton Rd, Atlanta, GA, 30322, USA; g Department of Reproductive, Family and Population Health, School of Public Health, Addis Ababa University, Zambia Street, Tikur Anbessa Hospital Building, Lideta Sub-city, Addis Ababa, Ethiopia; h Carolina Population Center, University of North Carolina at Chapel Hill, 123 W. Franklin St, Chapel Hill, NC, 27516, USA; i Department of Maternal and Child Health, Gillings School of Global Public Health, University of North Carolina at Chapel Hill, 135 Dauer Dr, Chapel Hill, NC, 27599, USA; j Yale School of Public Health, Yale University, 60 College St, New Haven, CT, 06510, USA; k Bill & Melinda Gates Foundation, Seattle, USA

**Keywords:** Maternal health, Healthcare quality, Cultural safety, Childbirth, Ethiopia, Qualitative

## Abstract

Access to maternal health services has increased in Ethiopia during the past decades. However, increasing the demand for government birthing facility use remains challenging. In Ethiopia's Afar Region, these challenges are amplified given the poorly developed infrastructure, pastoral nature of communities, distinct cultural traditions, and the more nascent health system. This paper features semi-structured interviews with 22 women who were purposively sampled to explore their experiences giving birth in government health facilities in Afar. We used thematic analysis informed by a cultural safety framework to interpret findings. Our findings highlight how women understand, wield, and relinquish power and agency in the delivery room in government health facilities in Afar, Ethiopia. We found that Afari women are treated as ‘others’, that they manipulate their care as they negotiate ‘cultural safety’ in the health system, and that they use trust as a pathway towards more cultural safety. As the cultural safety framework calls for recognizing and navigating the diverse and fluid power dynamics of healthcare settings, the onus of negotiating power dynamics cannot be placed on Afari women, who are already multiply marginalized due to their ethnicity and gender. Health systems must adopt cultural safety in order to ensure health quality. Providers, particularly in regions with rich cultural diversity, must be trained in the cultural safety framework in order to be aware of and challenge the multidimensional power dynamics present in health encounters.

## Highlights

• Cultural safety is a powerful framework to interpret and contextualize minority women's experiences in birth.
• Afari women are treated as ‘others’ in their health system.
• Afari women manipulate their care to enhance their own safety during childbirth.
• Health systems, and their providers, must adopt cultural safety to ensure health quality.

## 1 Introduction

### 1.1 The global transition from care access to access and care quality

Access to health-center and hospital-based maternal health services has significantly increased over the last few decades in low- and middle-income countries (LMICs) (Victora et al., 2016). The focus has shifted from healthcare delivery alone to healthcare systems to be more responsive to individual and community expectations and increase the social value placed on the care they offer. In 2018, Kruk et al. called for a revolution of health systems and the Lancet Global Health Commission on High Quality Health Systems rallied for equitable distribution of services, increasing the confidence of people using their health system, and providing consistent competent care and positive user experiences (Kruk et al., 2018). Moreover, the WHO and Organization for Economic Co-operation and Development and the World Bank published a report in 2018 that emphasized that quality improvements are needed, emphasizing how care quality is now at the forefront of global efforts to improve health care (Kieny et al., 2018). Indeed, women, and their children, suffer from persistent inequities related to systemic failures of health systems, facilities, and providers ranging from inaccessible services or providers' inflexibility and inability to respond or elicit patient preferences (Bohren et al., 2015). Many of these key domains of health care quality (e.g., dignity and respect, communication and autonomy, and supportive care) (Afulani et al., 2017) are intimately shaped by an individual's ability to access healthcare that they believe is tailored and responsive to their specific needs (Abebe et al., 2020), which are deeply shaped by their culture, context, and past health experiences (Larson et al., 2014). In this article, we extend research on quality of maternity care focusing on how women experience domains of communication, respect, and autonomy when the health system is built in a cultural system and history distinct from their own and power dynamics obfuscate their ability to express their needs.

Despite increased access to government birthing facilities in LMICs, incentivizing facility-based births may remain insufficient given that many communities prefer the comfort and ‘known’ components of ‘traditional’ birth approaches (e.g., birthing in the home, using assistance from a traditional birth attendant) (World Health Organization, 2017). Recent responses to demands for more equitable care may be best served with a focus on populations that contend with what macro level institutions define as ‘safe maternal and newborn care’ vis-à-vis their own constructions of high-quality care (World Health Organization, 2017). Some families may face additional constraints in accessing high-quality care if they engage in practices that do not fit into the quality care guidelines. For example, women that require additional care for previous female genital cutting (FGC) may not obtain the care they ask for (M. o. H. Ethiopia).

Kruk et al. (2018) call for high-quality health systems that emphasize the cruciality of people-centeredness to address power and information imbalances between providers and patients. Health systems must empower patients to become system accountability agents. Their proposed framework draws on health systems and quality improvement frameworks with an explicit focus on processes of care and quality impacts of care ‘for people’. Key aspects of these domains are competent care and systems, positive user experience, confidence in health systems, and better health. However, these components are deeply complex and often vary depending on a woman's, and her community's, emotional, physical, and cultural needs throughout pregnancy and childbirth. In past qualitative work exploring experiences of childbirth in Ethiopia, the authors found that the power some women wielded in the delivery room greatly depended on their own social and economic background along with the experiences and expectations they brought to their delivery (A. Hagaman et al., 2022). These multifaceted domains highlighted as crucial building blocks to revolutionizing health care quality for the majority of our world's population must be unpacked in context. The cultural safety framework is a useful lens to reinterpret and, ultimately, roll out the provision of ‘quality care’ in contexts where country biomedical and western-derived health systems may be foreign or incongruent to indigenous preferences and practices.

### 1.2 Cultural safety and maternal health care

Traditional approaches to cultural awareness and competency fail to adequately account for power relationships that are historically unequal between migrant or indigenous groups and healthcare providers and services. Cultural safety builds on and transcends the frameworks of patient centeredness, cultural sensitivity, and cultural competency by focusing on remediating power issues between patient and provider. While cultural humility and structural competency address the structural and ongoing processes of power dynamics in health care, cultural safety is the only framework that gives patients the authority to say whether or not they experienced a safe health encounter (Foronda et al., 2016; Metzl and Hansen, 2014). High-quality health systems care and person-centered maternal care (PCMC) call for patient needs to be centered and also assume that patients have enough information to ask questions about or challenge their care and are empowered to make decisions. Cultural safety, by contrast, highlights how ongoing power dynamics can preclude interactions that center the needs of patients who cannot express their needs for a variety of reasons (Papps and Ramsden, 1996). Unlike other frameworks that call for awareness of cultural differences between patients and providers, cultural safety centers providers’ ongoing navigation of power structures that began with colonization with the goal of treating all patients equitably (Ramsden, 1992). Since become prominent in other contexts, most prominently Australia and Canada, and the UK(Lokugamage et al., 2021). Some midwifery contexts have adopted cultural safety as a guiding framework for implementing care, though it is still an emerging paradigm and tools for assessment and program evaluation are still being developed.

Curtis et al. suggest that cultural safety training must be linked to measurable health equity outcomes (Curtis et al., 2019). While a consensus of what cultural safety training should consist of across all contexts does not exist, certain values connect the various approaches that health systems have instituted. Most fundamentally, only patients can assess whether or not cultural safety is present (Ramsden, 1992). Implementing cultural safety includes engaging staff on all levels (not only providers), as well as institutional policies and practices at all levels of the health system (Curtis et al., 2019). Some healthcare systems have taught skills such as active listening, worked to ensure adequate interpreters are available for patients, conducted trainings around racism in different contexts, and taught skills of diagnosing across various skin tones as part of applying a cultural safety framework (Abebe et al., 2020). Some of these strategies are also linked with a cultural humility framework; others, such as the Yagembeh-developed GY tool, are focused specifically on cultural safety. Other strategies include extending definitions of expertise. For example, during a recent training program in Colombia, medical students were taught about traditional medicines in a way that destigmatized them, which left them more open to incorporating patients' traditional medicines and understanding patients as experts about their own bodies. It also helped them acknowledge such practices as a part of a shared heritage with patients (J et al., 2021). Curtis also proposes using a provider's commitment to cultural safety as a health outcome. Therefore, in contexts where ‘culture’ is complex (multiple mother tongues, religions, health paradigms, and expectations of providers and institutions of ‘healthcare’), cultural safety is a powerful tool towards high quality care (Curtis et al., 2019). Several scholars have responded to critiques of cultural safety being too vague by developing tools for measuring and tracking it in healthcare encounters (Allwright et al., 2019). Additionally, some research has focused on showing how cultural safety is related to more familiar understandings of health outcomes, such as morbidity and mortality (Sarmiento et al., 2022).

### 1.3 Ethiopia maternal health overview

Health care access in Ethiopia has improved during the past two decades. In 2000, only 27% of Ethiopian women received antenatal care from a skilled provider; in 2019, this number had increased to 74%“ (Organization, 2015). In 2004, the Ethiopian government began expanding decentralized health services and increasing access to primary care and has been relatively successful. For example, the maternal mortality rate (MMR) decreased from 871 to 412 per 1000,000 live births between 2000 and 2016. During the same period, the neonatal mortality rate (NMR) decreased from 49 to 29 per 1000 live births. Despite this progress, Ethiopia's maternal and neonatal mortality rates have plateaued at a 10% reduction since 2015 (Ayele et al., 2021) and attaining continued increases in demand for services has remained challenging (Organization, 2015). As such, research is needed to understand gaps in the provision and experience of care (Ayele et al., 2021; F. D. R. o. Ethiopia & HEALTH, 2006; Victora et al., 2016).

While the Afar people make up over 90 percent of the population of the Afar Region, they are only 1.7 percent of the Ethiopian population (Federal Democratic Republic of Ethiopia Population Census Commission, 2008). The majority of Afar people are pastoral and travel according to the needs of their livestock (Central Agricultural Census, 2003). This has meant they have historically had less access to health resources because the health system cannot accommodate their mobility (F. D. R. o. Ethiopia & HEALTH, 2006). Despite the increase of health care access across Ethiopia, Afari people have relatively less access due to uneven distribution of skilled health workers (SHWs), which can also affect quality of care and patient satisfaction (King et al., 2015). According to the 2016 Ethiopian Demographic Health Survey (DHS), Afari women have access to comparatively lower levels of education and literacy (with high gender inequities), higher levels of poverty, and worse health outcomes compared to other districts (Central Stastical Agency, 2017). The Afar Region is known to have harsh environmental conditions such as extreme heat and drought, sometimes cited as possible obstacle to health care (King et al., 2015). Afari women also experience special obstacles to health care due lack of government investment in infrastructure, including language barriers from government providers. Many women continue to not access antenatal care and children frequently do not have access government-provided vaccine programs, despite state efforts to reach other rural region (Woldu Anbesu et al., 2021). Health facility birth often contradicts Afar tradition, because of religious beliefs about touching reproductive organs and the inability to perform culturally important practices around birth and death, such as delivering at home (Yousuf et al., 2011). It is also less costly and less time consuming to give birth at home, as one does not have to leave home and associated duties of caring for one's family and livestock (Henok and Takele, 2017). Of course, such barriers are shared among many communities in Ethiopia that limit their utilization of healthcare services.

Moreover, most Afari women have experienced some form of FGC, defined by the DHS as “removing some of the clitoris or the labia for nontherapeutic reasons”. FGC may complicate childbirth as it often requires access to a higher level of obstetric care (World Health Organization, 2023). According to the 2016 Ethiopian Demographic and Health Survey, the percentage of women with FGC in Ethiopia is 65%, though there are larger regional variations in prevalence and severity (e.g., some flesh removed vs. complete infibulation). For example, in the Afar Region, 98.4% of women undergo FGC. Most of these women (71%) are sewn closed in addition to being cut and having flesh removed, a practice known as fibulation. There is variation in beliefs among men and women if the practice should be continued, where Muslim women and women from rural areas are more likely to believe it should continue and women who have experienced FGC said it should be continued (Central Stastical Agency, 2017). According to the 2016 Ethiopian Health & Demographic Survey, 91.5% of women who experienced FGC in Afar were under 5 years and 68.3% of Afari women said the practice should be continued (Abebe et al., 2020; Central Stastical Agency, 2017). Guidelines put forth by a group of Ethiopian physicians state women with FGC should be first counseled and then deinfibulated during the second or third trimester. If they arrive at a health facility in labor while still infibulated, they should be examined, educated, and deinfibulated by a clinician trained in the procedure if a safe delivery would not be possible. Moreover, these guidelines state that no women should be reinfibulated by clinicians due to possible additional complications, though they do note the absence of clear national legal or policy statements. Instead, women should be counseled to not perform FGC on their daughters and to not seek reinfibulation for themselves (Gudu, 2016).

In this paper, we explore how women in the Afar region navigate Ethiopian medical infrastructure during childbirth. Thus, we extend the quality of care focus to center patients (particularly birthing women) as they interpret and narrate the quality of care they received in pastoralist Ethiopia. This paper adds to the growing scholarship on cultural safety in low-income settings, particularly those with historic geographic and cultural disparities in access to health care. Ultimately, we seek to inform the delivery of high-quality, patient-centered care in culturally diverse contexts where both there are growing investments in health care infrastructure and quality improvements in care delivery.

## 2 Methods

### 2.1 Study setting

The primary health unit of Afar consists of one zonal-level hospital and catchment health centers and health posts. The Afar Regional Health Bureau has a total of six hospitals, 86 health centers, and 379 health posts. In 2003, Ethiopia launched a national health extension program that was extended to pastoralist regions in 2006, increasing community health workers to link women to government healthcare. Due to shortage of female high-school graduates in the pastoralist regions (Afar and Somali) the health extension workers are male, unlike the agrarian regions where they are nearly exclusively female. Fieldwork was conducted between April and May of 2019 in Amibara, located in southern Afar region because of its geographic accessibility to our data collection teams (based in Addis Ababa). While the southern region of Afar is home to pastoral and semi-pastoral communities, it has also experienced increased population diversity and development, which is reflected through the mix of traditional Afar homes alongside stationary homes such as single-story structures and multistory apartment buildings. The town of Awash is a “central community hub” in Amibara, due to its location on the busy commercial highway, which runs to and from Djibouti, and its location adjacent to the Awash River. Participants eligible were women aged 18 and above who lived in Amibara and had delivered a live newborn within the past six-months at a government facility.

### 2.2 Parent study

This qualitative study was embedded within a mixed-methods evaluation of a health-systems quality improvement intervention implemented by the Ethiopian Ministry of Health and the Institute for Healthcare Improvement Ethiopia alongside evaluation partners at the Gillings School of Global Public Health, University of North Carolina at Chapel Hill and Addis Ababa University (Ethiopia). The study sought to ascertain how women experienced and perceived their interactions with MCH services they received at government facilities. The intervention was aimed at health staff and used a systems-embedded quality improvement (QI) approach (e.g., led and implemented by the Ethiopian Ministry of Health and health facility staff) to administer additional training in MHC best practices, QI training, and QI coaching to implement locally derived and cost-effective solutions to identified gaps in care, as well as to improve data quality for health information systems. The details of the nationally integrated intervention and subsequent findings are published elsewhere (Hagaman et al., 2022; Hagaman et al., 2020; Magge et al., 2019; Mengistu et al., 2021). In brief, the intervention improved data quality, safe child birth practices, and in some areas, respectful maternity care (Hagaman et al., 2020). Afar as a study site was intentionally included as a part of an equity-intentional design to better understand how the health system can serve Afari women in the context of low healthcare utilization and worse health outcomes. Qualitative data in this analysis was generated from four baseline study sites collected between [dates].

### 2.3 Sampling and recruitment

We purposively sampled 10 women who had experienced government health center (HC) deliveries and 10 who had delivered at government hospitals using delivery registries, delivery date, and location of birth. We did not purposively select women based on ethnicity (Afari and non-Afari (e.g., Amhara, Wolayita, etc). We anticipated that 20 interviews across HCs and Hospitals would be enough to reach thematic saturation based on our previous qualitative work in other diverse regions of Ethiopia (A. Hagaman et al., 2022; Hennink et al., 2019). Because we were advised by [x] in Afar that we would face challenges locating eligible women from a single health center due to their pastoral lifestyle, we recruited at three health centers: Andido, Awash, and Werer. Ten women from each HC were added to the sampling list. Muhammad Akle Hospital is the only hospital in Amibara and provided a large sampling pool, of which 30 were added to a sampling list.

Approval for community entry and data collection was achieved through communication between the fieldwork team and zonal officials, leaders from the Amibara Woreda Health Office and health facility administrators. Health extension workers (HEW), health facility staff (doctors, nurses, midwives, and record keepers and community residents) assisted the team in identifying and locating participants.

Ethiopian maternal healthcare implementation experts within IHI Ethiopia and the Ethiopian Ministry of Health advised us that the Andido HC would provide women that most closely reflected Afari women in Amibara. Awash HC provided women from communities that lived near the “main road” in relatively urban and developed parts of Amibara. Werer HC provided care to women who represented semi-pastoralist and pastoralist women that lived in an agricultural community in Amibara, where an agricultural research center is located. Among the women who delivered at Muhammed Akle Hospital, some lived near the hospital and others were referred from an HC.

### 2.4 Data collection

Between April 19 and May 5, 2019, two female Afari interviewers conducted 22 audio recorded interviews with 12 women from health centers (four women from Andido, Awash and Werer HCs, respectively) and 10 women from Muhammed Akle Hospital. Fifteen interviews were conducted in the Afar language; 7 were conducted in Amharic. The interviewers are sisters in the study site who are fluent in the Afar language, in Amharic, and in English. Because of their language proficiencies and familiarity with various communities in Amibara, the interviewers provided critical input to data collection and communication with participants and the research team.

Interviewers underwent a two-day training in qualitative interviewing methods and project orientation in Awash. Two study coordinators from Addis Ababa Univeristy [ASE adn DWK] and a research coordinator [HGR] from at Chapel Hill stayed with the interviewers to support them with data collection, interview debriefs, data management, and early analysis during the first week of interviewing.

The interviewers used a semi-structured interview guide built around three domains: pregnancy experiences (past and most recent), healthcare use (antenatal, delivery and postnatal), and satisfaction with MHC services. CB, ASE, DWR, AH, and HGR had jointly developed the original guide to assess experiences and satisfaction with MHC among women in Oromia and SNNPR in March 2018. For the study in Afar, updates included: asking about past and most recent pregnancies, directly asking women about their expectations for MHC and revising questions to maximize data saturation in key domains of provider treatment, communication, and other core domains within health quality. Interviewers also collected basic demographic information from participants, such as age, education, household size, religion, number of live births, and if/how often they visited a health facility throughout their pregnancy to receive ANC. Due to concerns over participant confidentiality and privacy, interviewers were not accompanied by local health facility staff when approaching women to participate in an interview, although they relied on the staff's knowledge to locate sampled women. Similarly, the interviewers did not allow others to be present during participant apart from very young children. They conducted the interviews in private spaces such as open fields, inside a parked vehicle of someone of the research team, the participants' homes, or homes of relatives. Following interviews, interviewers debriefed with study coordinators and wrote field notes in English separately. Each interviewer transcribed and translated their interview audio to produce verbatim transcripts in English. Transcriptions and translations were quality checked by the research team, with discussions to select the most appropriate interpretation at regular research team meetings.

### 2.5 Data analysis

Data were analyzed using NVivo. A team-based approach was used for iterative data collection and analysis. Team-based debriefs and field notes focused on key themes, observations, possible revisions to the interview guide. Additionally, some field notes included information that had not been captured during the recorded interviews that pertained to the domains at hand. The authorship team engaged in data triangulation, regularly engaging field notes, observations, and context specific expertise with the transcripts during analytic meetings. Broadly, we engaged in thematic analysis allowing for a flexible theoretic and analytic scope. Epistemologically, we treated the birthing mothers' experiences as real and true to them and at the same time, understand that they are co-constructed by their social environments and our qualitative interview process (Carter and Little, 2007). Through deep readings of the field notes and interview transcripts and analytic team meetings, a codebook was developed with both inductive and deductive approaches. Deductively, we coded elements of the mothers' narrative according to quality care determinants outlined in the theoretic frameworks referenced in the introduction (e.g., Donabedian and Kruk) as well as structural codes to delineate temporal windows of her narrative (e.g., antenatal, childbirth, postnatal). We identified inductive codes drawing on in-vivo coding, idioms and euphemisms, and repetition-based theme analysis. Multiple team members [AH, ECR, HC, MZ, CB, and HGR] applied codes to the textual data, initially dual coding transcripts with iterative team meetings with DWK and ASE to assess consistency in code understanding and application. Disagreements were discussed and adjustments made to the codebook. Thick descriptions of codes and themes were written and discussed during analytic meetings with the full authorship team. Theme development, which was led by the first author in consultation with co-authors, initially centered on identifying how Afari women comparatively described satisfaction and respect during healthcare encounters vis-à-vis their non-Afari counterparts. Thematic analysis then emerged, through our iterative analysis and engagement with relevant literature, as more specifically focused on how women experienced ‘cultural safety’. Thus, we used the cultural safety framework to interpret the core themes that emerged from our analysis. Results were written with quotes translated into English to illustrate our key findings. All mentions of respondent names are pseudonyms to maintain confidentiality.

### 2.6 Reflexivity statement

Our team consisted of Ethiopian and American academics (trained in anthropology, public health, and history of medicine), Ethiopian public health and medical professionals, and female Ethiopian public health researchers, all of whom related to this project differently. As outsiders all of us had cultural distance, with extremes among Americans and less among Ethiopian scholars and research assistants as their identities, life experiences, and shared cultural knowledge afforded more closeness and understanding of the nuances of women's lives. The authors that led the analysis and drafted the first version of this manuscript are two white women in the American academy with substantive methodological expertise in maternal-child health and qualitative methods; however, we were mindful to challenge our assumptions by staying close to our data, researching contextual details, and inquiring of our collaborators when we lacked information. We consider this project a collaboration toward situated knowledge rather than assertion of objectivity.

### 2.7 Ethics

Participants provided oral informed consent prior to any data collection. This research was determined as exempt by the University of North Carolina at Chapel Hill Institutional Review Board and obtained a program evaluation waiver from the Ethics Committee of Ethiopian Public Health Association. All methods were conducted according to the protocol outlined in the ethical approval application.

## 3 Results

A description of our sample can be found in Table 1. We interviewed 22 women between the ages of 15–37, the majority (68%) identifying as Afari ethnicity and Muslim religion (82%). Women reported between 1 and 7 live births and 55% delivered their last child at a government Health Center. Our findings highlight how women understand, wield, and relinquish power and agency in the delivery room in government facilities in Afar, Ethiopia. We describe how women's Afari ethnicity deeply shapes the cultural safety they have (or do not have) access to and the inequities and clinical tensions they face despite clinicians' efforts to provide care in resource strained environments. We first anchor our findings in the story of Malika, an Afari mother that shares the ways she was forced to wield agency in her own care, her disappointed and angered experiences of the health staff, and how the clinical care offered ‘does nothing’ for her or Afari mothers in her community. Malika's story is an extreme example of the physical risks minority women face in a health system shaped by her country's western biomedical health system. We then describe these dynamics, including how autonomy can be both empowered and required, forcing some women to manipulate their own care as they encounter lack of cultural safety that poses physical and emotional risk. We highlight how clinician's trust building can powerfully change maternal perceptions, and, finally, what health providers in culturally dynamic communities can do to address some of the issues illuminated by these women's experiences with facility based maternal healthcare.

**Table 1:** Participant characteristics.

	Age	# of live births	Delivery Location	Ethnicity	Religion	Education
1	28	4	Health Center	Amhara (non-Afari)	Muslim	Cannot read or write
2	20	1	Health Center	Afar	Muslim	Cannot read or write
3	35	2	Health Center	Unknown	Christian	Eighth grade
4	26	3	Health Center	Wolayita (non-Afari)	Protestant	Cannot read or write
5	26	6	Hospital	Afar	Muslim	Cannot read or write
6	20	4	Health Center	Afar	Muslim	Third grade
7	24	3	Hospital	Afar	Muslim	Eighth grade
8	37	7	Health Center	Afar	Muslim	Cannot read or write
9	36	7	Health Center	Afar	Muslim	Cannot read or write
10	25	1	Hospital	Afar	Muslim	Cannot read or write
11	22	2	Hospital	Afar	Muslim	Cannot read or write
12	20	2	Health Center	Afar	Muslim	Cannot read or write
13	26	5	Hospital	Afar	Muslim	Fifth grade
14	28	2	Health Center	Amhara (non-Afari)	Muslim	Tenth grade
15	20	1	Health Center	Afar	Muslim	Cannot read or write
16	25	6	Health Center	Afar	Muslim	Cannot read or write
17	20	1	Hospital	Wolayita (non-Afari)	Protestant	Fifth grade
18	20	2	Health Center	Wolayita (non-Afari)	Unknown	Cannot read or write
19	35	4	Hospital	Amhara (non-Afari)	Muslim	Cannot read or write
20	20	1	Hospital	Afar	Muslim	Cannot read or write
21	18	1	Hospital	Afar	Muslim	Fourth Grade
22	20	1	Hospital	Afar	Muslim	Tenth grade

### 3.1 Case study: malika, 26-year-old afari mother with five live births

Malika was 13 years old when she delivered her first child. At 26 years, for her fifth and most recent birth, she attended her local health center for antenatal care and was told her baby was not in a ‘good position,’ and also told the issue would resolve itself soon. When nine months pregnant, she visited the health center again and the baby did not move, then was told to come back when she went into labor. Malika was disappointed the staff did not ‘do more’ for her, and demanded to be transferred to the hospital, fearing complications. The staff agreed, and while in labor at the hospital, she had to actively take a role in her own delivery after nurses told her to “keep pushing” but she could not deliver her baby because of the vaginal closure due to FGC. She felt the nurses continued to “do nothing else to help” her. Malika, having a strong awareness of her own needs, sensed her baby was losing oxygen with health staff doing nothing, she performed a deinfibulation on herself

I was holding a knife and I cut my vagina myself. [Interviewer: you cut it yourself? [shocked]] why not? [mother clapping her hand] … I cut all the parts and the birth attendant came and cut the placenta and left. I told her if she couldn’t cut it for me I was going to use silk and put it in the placenta then cut it using scissors with my own hands. I then pushed to remove the leftover parts of the placenta … I gave birth by my own hands to save my life.

Afterwards, nurses left her alone in the small delivery room. Malika believed that the actions she took to perform care on herself saved her own life during childbirth. In the hours following delivery, she demanded again to be moved to a higher level hospital so her baby could be treated, as she sensed she was “in the state of losing her oxygen,” but as she tried to breastfeed to save her newborn's life, the child died on the way to a specialty hospital in Addis Ababa. Malika was angry and offended by the neglect and mistreatment she received. She shared that she saw other laboring women be mistreated as well. She summarized her opinions of the staff sharing, “I don't like their [health staff] work. They don't have heart [empathy] for people.“

Malika's story highlights several dynamics of care manipulation and the expectations and trust in health staff as crucial to cultural safety. Her culturally unsafe environment facilitated a loss of trust in the staff and the subsequent endurance of layers of physical and emotional violence. Malika expressed the necessity of her intervening in her own care to save both her and her child. This dynamic was echoed by several other Afari women, who often confronted health staff who perceived them as bad patients due to their culturally specific needs.

### 3.2 Afari women as ‘others’ in their health system

Like Malika, other Afari women reported approaching care in government facilities as a compromise they made to have a safe birth at the expense of giving up some of their care preferences. The lack of accommodation for Afari women's beliefs and practices repeatedly led to them having to manipulate their care in both subtle (lying or not disclosing health information) and overt ways (delivering her own placenta). One Afari woman reported lying about her physical health needs so she could be quickly discharged from the hospital within hours of her delivery. She did not want the male to use his hands to stop her postpartum bleeding (Kadiga, Afari (Ethnicity), 20y (age in years), 4 live births (LBs). She explained,

I: “Then he [health provider] argued with me, asking me why I came to the center if I didn’t want his help. So then I closed my legs together really fast and hard [demonstrated this by clapping her hand together quickly and loudly]. And then I stood up from the bed and left the room.”

I: By yourself ?

P: Yes

I: You told him that’s enough?

P: Yes, I told him I didn’t want him to do that, and I don’t want to come back to the center.

Despite this experience, the woman said she would continue to use a government health center for future births. She elaborated that she would attempt to use a different health center, but that “I like to be treated by health care. I don't think I will stop myself from going there and things happen with God's will.” Even though this woman received care that did not conform with her cultural beliefs and preferences, she believed the health center was the safest place to give birth. Similarly, multiple Afari women said they preferred not giving birth at a health center to avoid invasive digital penetration and excessive vaginal examinations, “In the hospital, everyone comes and puts their fingers in you. Women's fingers, men's fingers, everyone comes and puts their hands in you” (Halima, Afari, 22y, 2 LBs). Yet, they said they would return to the health center because of health concerns and lack of other safe options, “I don't have anywhere to go, so I will deliver in this place” (Kuhuli, Afari, 20y, 1 LB). These findings illustrate how Afari women chose to compromise their cultural safety to ensure their newborn's and their own physical safety.

The disconnect between health-center policies and Afari women's beliefs and practices sometimes led to them being constructed as bad patients by their non-Afari counterparts. For example, one Ahmara participant said she perceived that Afari women who followed traditional practices generally were unaware of healthcare facilities, did not understand basic health concepts such as the use of contraception, and resisted health advice that contradicted their cultural practices. When asked about how health staff treated their patients, the woman responded, “the local women do not understand anything … most of them are victims of female genital cutting and when the staff try to stich them after delivery the people here (Afari women) beat them, so the community has problems, not the staff” (Fatuma, Amhara (non-Afari), 28y, 4 LB). This woman implied that Afari women were aggressive toward the staff when they received delivery services because of their FGC practices.

Moreover, Afari women who received dissatisfactory and sometimes unsafe FGC care during delivery identified the ethnic discordance between them and their non-Afari provider and indicated that non-Afari health staff intentionally mistreated Afari women's needs related to FGC. Like Malika, one woman expressed anger that staff would not give her appropriate care (defibulation) that they knew she needed.

“They don’t cut the part to be cut and only tell you to push … in that time you become out of breath and your heart slows down … if mothers don’t have energy then they will die at that time” (Hawa, Afari, 26y, 5 LB).

During our interviews, only Afari women reported experiences of discrimination or culturally incompetent maternal health care by health staff, employers, and the health system.

### 3.3 Afari women manipulate their care as they negotiate ‘cultural safety’ in the clinic

Afari women discussed how they needed to advocate for, compensate for, or in some instances as we highlighted above, perform their own care to protect their physical, emotional, and interpersonal care needs. No non-Afari women describe manipulating the care they received. As Afari women tried to negotiate biomedical care approaches that were not built with their needs in mind, manipulation was used to protect themselves and meet their needs. Needs included specific additional care for FGC-induced scarring, limited digital penetration and touching of the uterus, preferring female providers, and agency to include traditional practices during delivery. Some of these experiences are linked to clinical mistreatment and failure to accommodate Afari women's cultural preferences and physical needs. For example, Afari woman explained how traditional birth attendants (TBAs) treated their FGC needs after delivery that the health staff, “did nothing for”. After doing an episiotomy of the woman's FGC scar tissue, the health staff did not sew the area back up post-delivery. The woman instead sought traditional care in her home community from a TBA to sew the tissue back together (e.g., refibulation). She explained,

P: The part he [health staff] left, we put perfume on it and added some beans which is called *bure* and we put it as it was [mother clapping while talking].

I: you did it yourself?

P: yes. our experienced woman [TBA] came and she did it like this [showing her two legs by putting them together] and she did put the part together (Mayram, Afari, 36y, 7 LB)

The TBA provided appropriate traditional medicines and sewed the woman's genitals to “put it as it was”. Women relied on cultural healthcare providers (such as TBAs) to fill gaps in care left by the health facilities. Another example highlights an Afari woman manipulating her care to address her needs of manual removal of placenta after childbirth. Her nurse explained that she needed to wash the woman's uterus with her hand. Upon being questioned, the nurse elaborated that it was important for the woman's health to clean her uterus and that this procedure is the only option. A TBA accompanying this mother told her to listen to the nurse, but she still refused the nurse's intervention and instead asked the TBA to do it in her way. Her TBA then, “put her fingers in her umbilical cord scroll her hand from the one side like by doing circle then immediately after she did that her placenta was removed from her uterus” (Cate, Afari, 25y, 6 LB). While she does not provide reasons for her unwavering opposition to the nurse's care, her preference for the TBA indicates her increased comfort and trust in a woman who belongs to her community.

Some Afari women described more covert ways that they supplemented their care. One woman was sent home after arriving at the health center because her membranes had prematurely ruptured, an occurrence she called *ali*. She was told that the nurses “could not do anything” for her, and spent the next two days in pain before she finally delivered. During this time community members brought her water that they had blessed. She explained, “… they [other people in the village] bring water as a culture and they throw the water on your face or your abdomen. That water they used on me had been prayed over” (Makkiya, Afari, 20y, 2 LB). She reported that after this interaction, her pain subsided, her amniotic fluid stopped leaking, and her labor proceeded. At this time, she used an ambulance to travel to the health center to give birth. The inability of the health staff to initially address her premature rupture of membrane influenced the woman's decision to return home and receive traditional care for her delivery complication. She believed the traditional care she received stimulated the successful progression of her labor.

Afari women negotiated added burdens of monitoring, advocating and attending to their own care resulting in subtle manipulation (e.g., lying to staff to receive the care they needed) to extremes of defibulation and reinfibulation. They described neglect and lack of communication from their government facility providers that facilitated manipulation of their own care, and perceptions of mistreatment. For example, women described male staff putting their hands inside their uterus as improper, refusal to reinfibulate as incomplete care, and recommendations to labor at home as neglect. The lack of communication and unmet expectations of ‘proper’ care are some of the main drivers of inequities Afari women faced in the delivery room. When asked what she had expected from the staff during her delivery, one woman responded, “I didn't expect anything from the ….Nothing. My mom was the one who did everything and she did whatever I want” (Kuhuli, Afar, 20y, 1 LB).

### 3.4 Trust as a pathway towards more cultural safety

Health system and health workers' trustworthiness was evaluated by women at every step of their care. Trust in health providers was described as a dynamic relationship that is earned and lost throughout a woman's involvement in care. Women largely trusted the formal health system to provide technical skills and medical supplies assuring physical safety of the patient. Women described building trust in health staff and facilities by the recommendations of peers and positive personal experiences they had in the past. Many women described ambivalence or neutral trust, citing a lack of knowledge about what to expect of their maternal healthcare. Women lost trust in health facilities when they experienced miscommunication and adverse care they felt was offensive or endangered their health. Women's trust in health facilities and staff shaped their propensity to (not) follow health advice and their selection of future health care facilities.

Many women who had few or no previous pregnancies or had previously only given birth at home described how they had implicit trust that the facility would deliver their babies safely, but they did not know what to expect of staff. One woman explained that she did not have any expectations, but trusted the health system to detect urgent problems. She explained: “I use the center because of the health of myself. … and if we are pregnant we don't know if the baby is not in good position or not … for those reasons I will use the center, but I don't expect anything from them [staff].” (Makkiya, Woyalita, 20y, 2 LB). This woman describes trusting the facility but withheld comments about their perceptions of staff trust. Another woman expanded on this same point when asked if she believed in the knowledge and skill of the staff, “yes I do believe, I did not notice them that much but they are good. I don't judge them because I don't know how they should work, but all I know is that they treated me well” (Ayisa, Afari, 37y, 7 LB). Due to her lack of expectations, she did not feel capable to comment on the technical capacity and knowledge of health staff. Women with low parity, previous home births, or uneventful facility births typically articulated indifference in trusting health personnel because they have not had personal experiences with facility-based maternal healthcare that threatened their expectations of a safe delivery.

### 3.5 Processes of losing, building, and maintaining trust during delivery

Trust played an integral role in women's experiences of healthcare, and ultimately threatened or bolstered their sense of cultural safety and what they needed to do to optimize their own health, and the health of their newborn. Women withdrew or extended trust based on whether a sense of cultural safety (e.g., having a sense that their values and preferences were recognized and supported), as well as physical and emotional safety, was present for them or not. Trust, therefore, was a salient indicator of a culturally safe, or unsafe, environment.

Despite Afari women entering the health center with disadvantages related to their complex health needs relating to FGC and incongruent cultural expectations, women did describe processes where trust was developed, creating a culturally safe environment for her to experience high quality care. A 28-year-old Ahmara woman said she expected to be treated well by staff and to have a healthy delivery. She elaborated that she trusted the staff's advice. She explained, “They give advice if you are the type who understands … proper treatment is their approach. Maybe you don't know where to register, especially when you are new. They show it to you, if they give you advice what more is left [for them to do]? Their service is good … the staff, they treat me well.” (Fatuma, Amhara (non-Afari), 28y, 4 LB). Both Afari and non-Afari women expressed some level of implicit trust in the health system, namely that it would provide them with necessary medical care. However, they differed in what aspects of the system they trusted. In contrast, Afari women expressed trust that the health center would be the safest place to deliver; they rarely spoke of positive interactions with the health staff. One woman noted the negative changes in health staff, “It was great. They gave us morale, gave us good face, their invitation was nice, and they told us what was good for us. They did their work properly. But when we see the staff now, *we* are the ones who call them, and *we* beg them to serve us … I don't have any trust.” (Asiya, Afari, 26y, 6 LB).

A 22-year-old with two children shared that she was disappointed in local health facilities after her first birth that resulted in a severe obstetric fistula. The woman explained how this happened, “they [health staff] hurt my body and cut me in many places … I was in pain and my body was in bad condition. In the hospital they didn't sew my body back, and they cut me and just played with me” (Halima, Afari, 22, 2 LB). She shared that this experience caused her to try to avoid getting pregnant again because she had no trust in maternal care assuring her physical or cultural safety. Despite this experience, she regained trust in the medical system during her most recent delivery. The health staff were attentive to her needs and competently managed her delivery explaining, “it was good. They are good when it comes to pregnancy. I don't know what they do to other women, but for me, they were with me. The girls [health staff] didn't leave me, they were with me the whole time.” She explained that their continuous support during labor and delivery was emotionally and physically helpful. She did, however, consider that her care might not reflect and extend to all women's care and, after all, “Afari women preferred home deliveries with traditional birth attendants.“

In all Afari women's accounts, their ethnicity was not directly referred to as a reason for the type and quality of care they received, but it was referenced often for what they *preferred* (that no hands entered their uterus, that their FGC was deinfibulated and then reinfibulated, etc). Many of the complications and conflicts around their care that directly impacted the trust and confidence they had in their providers revolved around the management of their female genital cutting (FGC). Non-Afari women did not mention FGC.

### 3.6 Towards culturally safe care: the role of health staff

Afari women, and women that experienced FGC, do not enter the government health facility on the same ground as patients without these complex conditions. As women described above, they often had to rely on their own agency to manipulate their healthcare to make it more culturally safe and acceptable for them. Women were asked to provide recommendations to the facilities and their staff, though they found it difficult to articulate what they needed exactly from staff. For example, one woman said she would recommend that other women use the health center but could not articulate why. When asked by the interviewer, she laughed and said, “I don't know, I will just recommend it” (Makkiya, Wolayita (non-Afari), 20y, 2 LB). While they might not know what to say, participants said health staff should be responsive to women's needs as they emerge during the antenatal care and delivery processes. Easier to articulate were recommendation for basic needs that health staff should provide, such as mosquito nets, water, shower, glucose, rest, and vaccinations. A woman with a history of six births said that health centers and hospitals should provide food for patients and their children, adding that she thought this was standard care in other regions, but not in Afar

“We really want to have some of the foods, but they don’t give it us. I don’t know why ….No one tells them [staff] to give the mother and children food. In this region, they exclude us. It’s not like other regions.” (Rahima, Afari, 26y, 6 LB).

There were moments of women experiencing culturally safe care, despite their presumably incongruent cultural needs and expectations with that of the biomedical health facility they entrusted their delivery with. One woman described how her mother and aunt were allowed to accompany her during her birth. “I asked them if my mom could be with me, and they did what I wanted” (Jemila, Afari, 20y, 1 LB). Women said that their preferences, when articulated and fulfilled, wielded positive reflections in her birth narration.

A lack of cultural safety and subsequent loss of trust in the health system left women vulnerable to not returning to health centers and hospitals for subsequent births. Malika was forced to perform her own defibulation, an event that emphasized an environment that was both physically and emotionally unsafe for her and that did not meet her culturally specific needs. She had chosen to come to a health center to give birth hoping for a good outcome for her and her baby and even had advocated for herself to be transferred to a hospital when her birth became complicated. However, after the health staff's resistance to performing defibulation and after she and her baby were not transferred quickly enough to a higher level hospital, she felt that her self-advocacy had not accomplished anything. Moreover, her baby was dead. The lack of cultural safety Malika experienced in the health center and the hospital caused her to lose the little trust she had in the health system.

For some Afari women, culturally safe care includes providing competent FGC care. Health staff should ensure that they understand women's expectations by discussing them in advance. For such important and sensitive care for FGC, women should be treated respectfully and nonjudgmentally in an open conversation to set realistic expectations and build women's trust. For example, many women expressed they did not expect physical and painful touching of their uterus. Some expressed they do not want male health care providers. Women described trust that the greater health system would hold individual facilities and providers accountable in instances of mistreatment. To maintain this trust in the healthcare system and ensure that women continue to use health facilities, the health system should ensure that there are effective mechanisms to ensure healthcare quality.

## 4 Discussion

We argue that Afari women, as an ethnic and cultural minority in Ethiopia, experience limited cultural safety in facility-based healthcare, where they face challenges to their cultural identity and must work hard to attain ‘safe deliveries’. Because of their marginal status which causes them to experience discrimination within an already strained healthcare system, Afari women must be aware of power dynamics in health encounters. Besides having to exert extra effort to ensure they receive quality health care, marginalized women carry the additional emotional and social burden required to navigate health encounters, because their cultural safety and well-being depend on their awareness of power dynamics.

We found that because Afari women are marginalized due to their culture and health histories (such as FGC), they experience less cultural safety in facility-based healthcare, where they face challenges to their cultural identity and who expressed discrimination their non-Afari counterparts did not discuss to attain the same ‘safe deliveries’. Afari women are forced to exert incredible effort than their non-Afar counterparts did not report while receiving health care. The quality of the care they receive depends on their awareness of structural power dynamics that influence what happens in health care settings. While Afari women trusted the health system's technical abilities, a lack of confidence that their cultural and physical needs would be met at every stage of ANC and birth left them vulnerable to not return to the health system for subsequent births. Afari women in the Afar Region are [forced] to exercise autonomy of care because of lack of communication with health care professionals. This highlights how trust between Afari women and health care providers must be built and is not static. Rather, trust is a tool Afari women use to negotiate their health care that can be extended and withdrawn.

The cultural safety framework posits that the patient is the arbiter of appropriate care and that healthcare system must develop tools to respond to patients’ needs. The onus of negotiating power dynamics cannot be placed on Afari women, who are already multiply marginalized due to their ethnicity, gender, and because they are pregnant. Providers must be trained in the cultural safety framework in order to be aware of and challenge the multidimensional power dynamics present in health encounters, such as the Bass Reflexivity Tool, active listening, and the Ganngaleh nga Yagaleh (GY) tool that assesses commitment and adherence to cultural safety developed by the Yugembeh People in Gold Coast, Australia (Bass et al., 2022; Lokugamage et al., 2021; West et al., 2021). Cultural safety does not call for merely learning about marginalized cultures, but rather gaining skills to recognize and navigate the diverse and fluid power dynamics of healthcare settings. This requires ongoing reflection on the part of the providers and at the level of the health center to ensure they are meeting the needs of patients (Ramsden, 1992) Providers, thus, have much to learn from women that experience cultural unsafety in the clinic.

Cultural safety highlights the many structural levels that contribute to equity or inequity in health. On the individual level, women expressed their desire for concrete improvements to the system that would improve their health: Afari women cited adequate mosquito nets, food, clean rooms as important to their physical safety while giving birth. The framework also underscores structural issues that require collaboration above the individual level. For example, despite criminalization of FGC in 2005 and falling rates of the practice, 65% of women in Ethiopia have experienced FGC. Rates are likely to be higher due to underreporting.^1^ Moreover, women in Afar are more likely to be married to men who support the practice, a reminder that decisions to participate are embedded in cultural norms and social networks rather than strictly individual decisions (Sara et al., 2022). Many of these women will need culturally safe care regarding this issue. This issue therefore extends beyond Afar. Cultural safety research highlights acknowledging culturally specific needs as a crucial first step to achieving cultural safety (Giles and Audrey, 2016). Our paper extends that research by highlighting how pregnant patients and their babies may be in more physical danger when their culturally specific needs are not met. In another study, patients cited respect for their choices and their own preferences for care as critical to cultural safety (Churchill et al., 2020). Despite FGC management recommendations including education and counseling on FGC management during pregnancy, delivery, and postpartum, women in our study never cited a provider explaining to them the defibulation process, a preference for having this procedure before delivery, or how to manage their post-birth care especially if there is a refusal to re-infibulate. Such conversations are needed early and often and should include a patient-led decision-making process.

Cultural safety can also be incorporated on a systems level through a focus on diversity and inclusion in administrative and hiring processes. A growing body of literature is focusing on how cultural safety can ameliorate health staff stress brought about by workplace discrimination (van Ryn and Saha, 2011) and imparts many of the same benefits in a staff setting as in a clinical setting (Lokugamage et al., 2021). This could address increasing health staff stress and burnout that contributes to lower quality health outcomes in LMICs (Afulani et al., 2021).

Our study has limitations. Our findings may not be representative of other parts of Ethiopia, because we purposively sampled women from one region. However, our study could provide transferable insights into other maternal care settings. Interview audio was first translated into Amharic, if conducted in a different language, to provide universal understanding among our Ethiopian team, some whom did not speak Afari, and then again translated into English. Language manipulation could have led to the loss of some linguistic nuances. Moreover, the intense subject matter of the interviews and our sampling methods could have led to some women feeling uncomfortable expressing distressing experiences, even though interviewers took care to build rapport, ensure confidentiality, and establish consent. Given data was collected through one in-depth interview, there may be various drivers of what, and how, participants chose to disclose challenging experiences with their births and health facilities. Finally, while our study engages cultural safety, we did not ask participants directly whether or not they felt culturally safe and how they experienced culturally safe or unsafe care.

## 5 Conclusion

Afari women described how they are treated as “others” in healthcare facilitates and often need to advocate for themselves, and in some instances, even address their care needs on their own. Furthermore, women directly attributed their distrust of healthcare providers and adverse birth outcomes to the lack of cultural safety they experienced in the birthing room. These results highlight the need for effective strategies to promote cultural safety and high-quality care for Afari women in Ethiopia and ultimately reduce inequities in maternal and child health.

## Data Availability

Data will be made available on request.
