# Supplementary material for: Navigating and manipulating childbirth services in Afar, Ethiopia: A qualitative study of cultural safety in the birthing room
Source: Soc Sci Med. 2023 Aug;331:116073. doi: 10.1016/j.socscimed.2023.116073 (PMC10410251; doi:10.1016/j.socscimed.2023.116073)
Supplement: Multimedia component 1 [file mmc1.docx]

**Supplemental File: Qualitative Interview Guide**

**Participant ID**: ______________

**Date:** _____________________

**Interviewer name:** _________________________

**Language of Interview:** _____________________

**Translator (if present):** ___________________

**Region**: ____Afar____

**Woreda**: _____________________

**Health facility name:** ______________________

PURPOSE: build rapport

1. To begin, I’d like to get to know you a little bit more. Tell me, what is it like for you being a mother?

- Tell me about your family.

PURPOSE: understand pregnancy experience

1. Tell me about your most recent pregnancy. How did you find out you were pregnant?

- Can you describe to me how you felt during your pregnancy?
- What emotions you feel throughout your pregnancy? Tell me more.
- How did you feel physically during your pregnancy?

1. Did anything concern you during your pregnancy? Tell me more about this….

1. Who gave you advice during your pregnancy?
   - What did people tell you?

1. Tell me about your past pregnancies… How did this pregnancy compare to past pregnancies?

- How did your past pregnancy experiences affect how you felt about this pregnancy?

PURPOSE: understand prenatal care experience

1. I would now like to learn about your experiences with prenatal care. Tell me, why did you begin prenatal services at the health center/hospital?

- How often did you come for prenatal care?
- Who, if anyone, helped you get care at the facility?
- What, if any, concerns did you have coming to this clinic/facility? Tell me more…

1. Describe one prenatal visit for me. Tell everything that happened during this visit from the moment you arrived until you left the health center/hospital.

1. How did health staff treat you during prenatal visits?

- Give me example of how they treated you.

1. Based on your prenatal visits, what did you expect for your delivery?

- What did you expect from staff at the health facility?

Purpose: understand delivery experience

1. Now I would like to learn about your delivery experience. Tell me about your delivery experience. Start from the moment you began laboring until you left the health center/hospital after giving birth.

1. SPACE: I do not know much about the health center/hospital you attended. Can you describe the space where you received services to me?

- What did you like about the space?
- What did you dislike about the space?
- How would you describe the cleanliness?
- What about privacy?

1. STAFF TREATMENT: Tell me about the staff at the health center/hospital. How did they treat you during your delivery? Tell me about this…

- Can you give me an example?
- How did that make you feel?

1. How confident did you feel in the knowledge and skills of clinic staff? Tell me more…

- If applicable: how does your experience with compare to prior deliveries at a health facility?

PURPOSE: understand postnatal care experience

1. Now I would like to learn about your postnatal care experiences after your baby was born. Did you receive any follow-up two days after delivery at the clinic or at your house?

- Explain the visit to me from start to finish…

1. What kind of follow-up did you expect to receive after your baby was born? Tell me more...

1. Does your community have a confinement practice/period after delivery? Tell me about this.

- What was your experience with confinement?

PURPOSE: understand overall satisfaction with maternal health services

1. How do you believe the health care you received helped you?

1. How do you believe the healthcare you received helped your child?

1. Based on your experiences, would you use these services again? Tell me more about why or why not.

1. Would you recommend these services to other pregnant women? Tell me more about why or why not…

1. How can the health center/hospital make the experience of childbirth better for women such as yourself?

- What is needed?
- Who should do this?

1. Thank you for sharing your experiences with me, I really appreciate your time.

- Do you have any questions or comments for me?
